# The Effect of Anterior Uterocervical Angle on Primary Dysmenorrhea and Disease Severity

**DOI:** 10.1155/2018/9819402

**Published:** 2018-09-17

**Authors:** Mefkure Eraslan Sahin, Erdem Sahin, Yusuf Madendag, Ilknur Col Madendag, Ahter Tanay Tayyar, Fatma Özdemir, Gokhan Acmaz, Iptisam Ipek Muderris

**Affiliations:** ^1^Department of Obstetrics and Gynecology, Health Sciences University Sivas Sarkışla Government Hospital, Sivas, Turkey; ^2^Department of Obstetrics and Gynecology, Erciyes University Medicine Faculty, Kayseri, Turkey; ^3^Department of Obstetrics and Gynecology, Health Sciences University Kayseri Education and Research Hospital, Kayseri, Turkey; ^4^Department of Obstetrics and Gynecology, Acibadem Maslak Hospital, Istanbul, Turkey

## Abstract

**Background:**

Primary dysmenorrhea, defined as painful menstrual cramps originating in the uterus without underlying pathology, is a gynecological disease that affects quality of life and school success. Our goal was to determine the effect of anterior uterocervical angle on primary dysmenorrhea and disease severity.

**Methods:**

A total of 200 virgin adolescents, 16 to 20 years of age, were included in the study. The Andersch and Milsom scale was used to determine dysmenorrhea severity. Those with pathologies causing secondary dysmenorrhea were excluded from the study. Study subjects were grouped based on severity of pain. Demographic characteristics and uterocervical ultrasonographic measurements were compared among groups.

**Results:**

Of the 200 participants enrolled in the study, 50 were healthy controls and 150 had primary dysmenorrhea. Those with primary dysmenorrhea had a significant family history of primary dysmenorrhea compared with controls (*P* < 0.001). Age (*P*=0.668), body mass index (*P*=0.898), menarche age (*P*=0.915), and length of menstrual cycles (*P*=0.740) were similar in all groups. The uterine corpus longitudinal axis, uterine corpus transverse axis, and uterine cervix longitudinal axis were also similar (*P*=0.359, *P*=0.279, and *P*=0.369, resp.). The mean uterocervical angle was 146.8 ± 6.0 in controls and 143.3 ± 7.3 in those with mild pain with no significant difference between the groups. In those with moderate pain, the mean uterocervical angle was 121.2 ± 7.3 compared with 101 ± 9.2 in those with severe pain, which was a significant difference. Additionally, there was also a significant difference in the uterocervical angle among those with mild, moderate, and severe pain (*P* < 0.001).

**Conclusion:**

Our results indicate that a narrower anterior uterocervical angle is associated with primary dysmenorrhea and disease severity.

## 1. Introduction

Primary dysmenorrhea (PD), often seen in adolescent girls and women during the reproductive period, is defined as painful menstrual cramps originating from the uterus without underlying pathology. It is a gynecological disease that affects quality of life and school success [1, 2]. PD is characterized by suprapubic pain that starts several hours before menstrual bleeding and lasts a few hours after the culmination of bleeding. Symptoms peak with maximum blood flow and continue for two to three days [3].

The underlying cause of PD is not yet fully understood; however, several factors are known to play a role in the etiopathogenesis. One such factor is increased intrauterine pressure. In women without dysmenorrhea during menstrual bleeding, the basal uterine tonus is below 10 mmHg, and there are three to four rhythmic synchronized contractions every 10 minutes. However, in women with PD, the basal uterine tonus exceeds 10 mmHg, and every 10 minutes, there are four to five contractions without rhythm and coordination. Pressure during contractions can reach 150 to 180 mmHg [4]. When the uterine pressure exceeds the systemic arterial pressure, anaerobic metabolites are released due to ischemia and these metabolites stimulate nerve fibers leading to dysmenorrhea [5].

The uterocervical angle (UCA), which is between the cervical canal and the uterus frontal wall, is a newly investigated ultrasonographic parameter. In the literature, it has been stated that ultrasonographically measured cervical length and UCA can determine the clinical function of the cervix. Recently, Dziadosz et al. reported that in the presence of a large UCA, uterine contents are directly and more easily displaced to the cervix [6]. In another study, Zebitay et al. reported that cervical length and uterine cervical volume were significantly associated with PD in virgin adolescent girls [7].

Menstrual pain which is associated with PD augments during maximum blood flow and uterine pressure increases exceeding the systemic arterial pressure. This process is associated with the length of the cervix, thus it is possible to assume that the pain occurs with the difficult excretion of endometrial tissues. The hypothesis is that if one considers that the excretion of endometrial tissues becomes more difficult, then the UCA is narrow. The presence of a narrow UCA may lead to PD. This study aimed at determining the effect of anterior UCA on PD and disease severity.

## 2. Materials and Methods

This study was conducted at Sivas Sarkisla Government Hospital in Turkey. The study was approved by the Ethics Committee of Cumhuriyet University (decision number: 2018-01/12) according to the Declaration of Helsinki. Study participants included virgin adolescents aged 16 to 20 years who were referred to the gynecology clinic for complaints of dysmenorrhea.

The Andersch and Milsom scale was used to determine dysmenorrhea severity. Study participants were questioned concerning each of the three categories: work activity, systemic symptoms, and need for analgesics [1, 8, 9]. They were then divided into four groups based upon their level of reported pain as follows: (1) control group, menstruation is not painful and daily activity is unaffected; (2) mild pain group, menstruation is painful but seldom inhibits normal activity, analgesics are seldom required; (3) moderate pain group, daily activity is affected, analgesics are required and give relief so that absence from work or school is unusual; and (4) severe pain group, activity is clearly inhibited, poor effect of analgesics, vegetative symptoms, and severe pain exists.

Patients were excluded from the study if pathologies causing secondary dysmenorrhea existed, including: fibroids, adnexal masses (abscess, ovarian cyst, and hydrosalpinx), gynecological operation history, pelvic inflammatory disease history, presence of urinary or other infections, uterine anomaly, oral contraceptive use, alcohol and cigarette use, or nonsteroidal anti-inflammatory drug use in the last two days.

**Figure 1:**
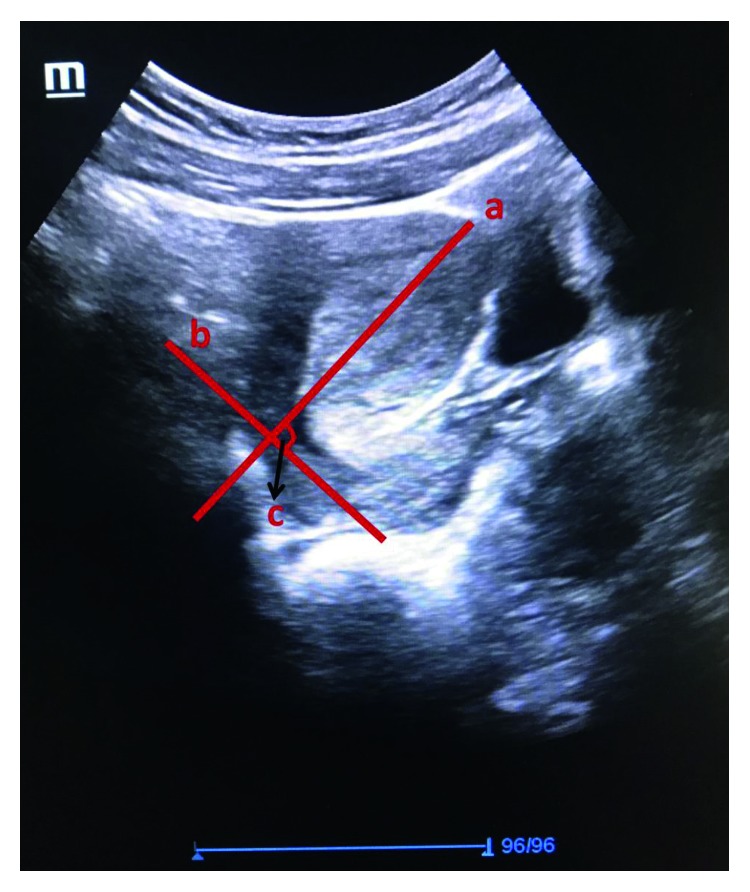
Ultrasonography measurements. (a) Uterine corpus longitudinal axis. (b) Uterine cervix longitudinal axis. (c) Anterior uterocervical angle.

The longitudinal and transverse axis of the uterine cervix and uterine corpus were measured after ablation by abdominal ultrasonography. Measurements were performed on the midsagittal plane by the same specialized person (MES). The uterine cervical longitudinal axis was measured when at least three of the following five recognition points on the plane were clearly visible: (1) the external cervical os, (2) the internal cervical os, (3) the cervical canal, (4) the cervical/vaginal interface, and (5) the cervical corpus [10]. When the length between the internal and external cervical os could not be measured on a flat plane due to the curvature of the cervical canal, the longitudinal diameter of the uterine cervix was measured as the sum of the possible linear measures taken in part. The longitudinal axis of the uterine corpus was measured on a longitudinal plane extending from the projection point of the endometrium on the uterine corpus to the internal cervical os where the internal cervical os and endometrium were visible the longest. The transverse axis of the uterine corpus was measured on the same plane perpendicular to the uterine corpus longitudinal axis where the uterine corpus was widest. The UCA was defined as the line drawn from the internal os to the external os and the angle formed by the second line passing through the internal cervical os parallel to the lower side of the anterior uterine wall in the internal os [11]. All measurements were performed on the second or third day of the menstrual cycle, and in order to minimalize intra and interobserver variabilities, all measurements were performed by a single sonographer (MES) using the same ultrasonography device (Mindray DC-7 Ultrasound System, China) and a 3.5 MHz abdominal probe (Figure 1).

To determine the number of patients evaluated in our study, we referenced the study conducted by Zebitay et al., entitled “Importance of cervical length in dysmenorrhea etiology” [7]. The Minitab®16 statistical program (Minitab Inc., State College, PA, USA) was used to perform the statistical analysis. The Shapiro–Wilk test was used to assess normality of the data, and Levene's test was used to assess for homogeneity of variances. Values are expressed as mean ± standard deviation. Parametric comparisons were made via a *t*-test or *z*-test, and nonparametric comparisons were made via a Mann–Whitney *U* test. A *P* value <0.05 was considered statistically significant.

## 3. Results

Of the 200 virgin adolescents enrolled in the study, 50 were healthy controls and 150 had PD. Demographic characteristics were compared and are shown in Table 1. Age (*P*=0.668), body mass index (*P*=0.898), menarche age (*P*=0.915), and length of menstrual cycles (*P*=0.740) were similar in all groups. Patients with PD had a significant family history of PD compared with controls (*P*  <  0.001).

Table 2 displays a comparison of ultrasonographic measurements among the groups. The uterine corpus longitudinal axis, uterine corpus transverse axis, and uterine cervix longitudinal axis were similar in both control and PD subjects (*P*=0.359, *P*=0.279, and *P*=0.369, resp.). The mean UCA was 146.8 ± 6.0 in control subjects and 143.3 ± 7.3 in those with mild pain, which was not significantly different. The mean UCA was 121.2 ± 7.3 in those with moderate pain and 101.9 ± 9.2 in those with severe pain, which was significantly different. Additionally, UCA was significantly different among those with mild, moderate, and severe pain (*P*  <  0.001).

## 4. Discussion

In the present study we aimed at determining the effect of anterior UCA on PD and disease severity. According to our findings, UCA was significantly narrower in patients with PD compared with those without, and lower UCA correlated with severity of disease. UCA seems to be an important anatomical factor in PD etiopathogenesis.

There is evidence in the literature that vasoconstriction-induced hypoxia has a role in the underlying pathogenesis of PD but the cause is not fully understood [7]. Numerous studies have focused on disease treatment but only one assessed the association of uterine and cervical anatomy with PD. In a study by Zebitay et al., they reported that the length of cervical longitudinal and transverse axis and uterine cervical volume significantly correlated with severity of PD [7]. In the present study, we did not find significant differences in the uterine corpus longitudinal axis, uterine corpus transverse axis, and uterine cervix longitudinal axis among the groups.

In the current study, we did find that narrower UCA is associated with PD and disease severity. Because the material in the uterine cavity will pass through a narrower structure than itself during the ejection of menstrual blood from the uterine cavity, the uterus is in need of a propulsive force due to the resistance that occurs in the form of friction. A narrower UCA will certainly increase this resistance. The relationship between this propulsive force and resistance can be explained by the Navier–Stokes equation, which is the basic motion equation for a viscous fluid, considering the unit volume of fluid, mass conversion, Newton's second law, energy conversion, and the second law of thermodynamics [7, 12]. In cases with a narrower UCA, an increase in uterine contractility may occur to overcome the greater frictional force present during the passage of blood. Parallel to the increased contraction, the UCA will produce a more intense pain sensation. This explains the significant correlation of UCA with pain severity in patients with dysmenorrhea.

In addition, in a prospective observational study over eight years, Juang et al. reported that both gestation length and type of delivery had an effect on PD. The most important improvement occurs after the first delivery. In addition, spontaneous vaginal delivery has the advantage of reducing the severity of dysmenorrhea compared with cesarean birth [13]. Although more prospective studies are needed, it can be assumed that the first birth reduces the severity of dysmenorrhea by changing the UCA and reducing resistance in the cervical canal. Other studies reported that the severity of dysmenorrhea was reduced by cervical dilatation in a recursive manner with artificial methods [4] and hot pad application [14]. The benefits of cervical dilatation in these studies have been explained by the reduction of resistance to cervical excretion due to the formation of a wider cervical canal after dilatation. This helps to reduce the severity of dysmenorrhea due to the reduction of friction and resistance, which decreases prostaglandin release and reduces uterine pressure [4]. These reports clearly show that the anatomy of the cervix is related to the severity of PD and disease. As such, they support the findings of our study.

However, there are some limitations in this study. Measurements were made with empty bladders to maintain normal anatomic position, which increased the difficulty of obtaining measurements.

## 5. Conclusion

The present study showed that a narrower anterior UCA is associated with PD and disease severity. Further prospective studies with a greater number of patients are required to further substantiate these results.

## Figures and Tables

**Table 1:** Comparison of demographic characteristics among groups.

	Group 1, control *n*=50	Group 2, mild pain *n*=50	Group 3, moderate pain *n*=50	Group 4, severe pain *n*=50	*P* value
Age (years)	17.60 ± 1.21^a^	17.50 ± 1.28^a^	17.68 ± 1.09^a^	17.42 ± 0.97^a^	0.668
BMI (kg/m^2^)	22.68 ± 1.22^a^	22.66 ± 1.14^a^	22.49 ± 1.07^a^	22.52 ± 1.05^a^	0.898
Menarche age (years)	12.74 ± 0.80^a^	12.82 ± 0.87^a^	12.78 ± 0.84^a^	12.68 ± 0.99^a^	0.915
Length of menstrual cycles (days)	28.22 ± 1.23^a^	28.40 ± 1.06^a^	28.34 ± 0.79^a^	28.42 ± 0.75^a^	0.740
Family history of PD *n* (%)	3 (6%)^a^	21 (42%)^b^	18 (36%)^b^	24 (48%)^b^	0 < 001

*Note.* BMI, body mass index; PD, primary dysmenorrhea. Superscript numbers indicate the absence (a) or presence (b) of statistically significant differences. The Minitab®16 statistical program (Minitab Inc., State College, PA, USA) was used to perform the statistical analysis. The Shapiro–Wilk test was used for normality assumption of the data, and Levene's test was used for the variance homogeneity assumption. Values are expressed as mean ± standard deviation. Parametric comparisons were made via *t*-test or *z*-test, and nonparametric comparisons were made via Mann–Whitney *U* tests. *P* < 0.05 was considered statistically significant.

**Table 2:** Comparison of ultrasonographic measurements among groups.

	Group 1, control *n*=50	Group 2, mild pain *n*=50	Group 3, moderate pain *n*=50	Group 4, severe pain *n*=50	*P* value
Uterine corpus longitudinal axis (mm)	45.60 ± 4.78^a^	44.76 ± 2.75^a^	46.08 ± 4.77^a^	45.94 ± 3.41^a^	0.359
Uterine corpus transverse axis (mm)	33.02 ± 2.41^a^	33.32 ± 2.18^a^	32.46 ± 2.13^a^	32.82 ± 2.25^a^	0.279
Uterine cervix longitudinal axis (mm)	31.94 ± 2.04^a^	32.10 ± 1.60^a^	31.66 ± 1.90^a^	32.46 ± 1.34^a^	0.369
Anterior uterocervical angle	146.8 ± 6.0^a^	143.3 ± 7.3^a^	121.2 ± 7.3^b^	101.9 ± 9.2^c^	0 < 001

*Note.* BMI, body mass index; PD, primary dysmenorrhea. Superscript numbers indicate the absence (a) or presence (b,c) of statistically significant differences. The Minitab®16 statistical program (Minitab Inc., State College, PA, USA) was used to perform the statistical analysis. The Shapiro–Wilk test was used to evaluate the normality assumption of the data and Levene's test was used to assess the variance homogeneity assumption. Values are expressed as mean ± standard deviation. Parametric comparisons were made via *t*-test or *z*-test, and nonparametric comparisons were made via Mann–Whitney *U* test. *P* < 0.05 was considered statistically significant.

## Data Availability

The data used to support the findings of this study are available from the corresponding author upon request.

## References

[1] Tomás-Rodríguez M. I., Palazón-Bru A., Martínez-St John D. R., Navarro-Cremades F., Toledo-Marhuenda J. V., Gil-Guillén V. F. (2017). Factors associated with increased pain in primary dysmenorrhea: analysis using a multivariate ordered logistic regression model. *Journal of Pediatric and Adolescent Gynecology*.

[2] Ortiz M. I. (2010). Primary dysmenorrhea among Mexican university students: prevalence, impact and treatment. *European Journal of Obstetrics and Gynecology and Reproductive Biology*.

[3] Burnett M., Lemyre M. (2017). No. 345-primary dysmenorrhea consensus guideline. *Journal of Obstetrics and Gynaecology Canada*.

[4] Dawood M. Y. (2006). Primary dysmenorrhea: advances in pathogenesis and management. *Obstetrics and Gynecology*.

[5] Altunyurt S., Göl M., Sezer O., Demir N. (2005). Primary dysmenorrhea and uterine blood flow: a color Doppler study. *Journal of reproductive medicine*.

[6] Dziadosz M., Bennett T. A., Dolin C. (2016). Uterocervical angle: a novel ultrasound screening tool to predict spontaneous preterm birth. *American Journal of Obstetrics and Gynecology*.

[7] Zebitay A. G., Verit F. F., Sakar M. N., Keskin S., Cetin O., Ulusoy A. I. (2016). Importance of cervical length in dysmenorrhoea aetiology. *Journal of Obstetrics and Gynaecology*.

[8] Ambresin A. E., Belanger R. E., Chamay C., Berchtold A., Narring F. (2012). Body dissatisfaction on top of depressive mood among adolescents with severe dysmenorrhea. *Journal of Pediatric and Adolescent Gynecology*.

[9] Ozerdogan N., Sayiner D., Ayranci U., Unsal A., Giray S. (2009). Prevalence and predictors of dysmenorrhea among students at a university in Turkey. *International Journal of Gynecology and Obstetrics*.

[10] Saul L. L., Kurtzman J. T., Hagemann C., Ghamsary M., Wing D. A. (2008). Is transabdominal sonography of the cervix after voiding a reliable method of cervical length assessment?. *Journal of Ultrasound in Medicine*.

[11] Knight J. C., TenBrink E., Sheng J., Patil A. S. (2017). Anterior uterocervical angle measurement improves prediction of cerclage failure. *Journal of Perinatology*.

[12] Chavez M. L., DeKorte C. J. (2003). Valdecoxib: a review. *Clinical Therapeutics*.

[13] Juang C. M., Yen M. S., Twu N. F., Horng H. C., Yu H. C., Chen C. Y. (2006). Impact of pregnancy on primary dysmenorrhea. *International Journal of Gynaecology and Obstetrics*.

[14] Akin M., Price W., Rodriguez G., Erasala G., Hurley G., Smith R. P. (2004). Continuous, low-level, topical heat wrap therapy as compared to acetaminophen for primary dysmenorrhea. *Journal of Reproductive Medicine*.

